# Influences of Land Policy on Urban Ecological Corridors Governance: A Case Study from Shanghai

**DOI:** 10.3390/ijerph19159747

**Published:** 2022-08-08

**Authors:** Xiaoping Zhou, Duanshuai Shen, Xiaokun Gu

**Affiliations:** 1 School of Government, Beijing Normal University, Beijing 100875, China; 2 China Institute for Urban Governance, Shanghai Jiao Tong University, Shanghai 200030, China; 3 School of International and Public Affairs, Shanghai Jiao Tong University, Shanghai 200030, China

**Keywords:** urban green infrastructure, ecological corridors, ecological space, land use change, land policy, planning, urban governance, metropolitan, Shanghai

## Abstract

The analysis of land use change (LUC) characteristics and the impact of policies related to urban ecological space is required to improve spatial planning and to support decision making regarding green infrastructure (GI) investment. This study employed Geo-informatic Tupu analysis and Fluctuation Potential Tupu analysis methods to analyze the characteristics of LUC in an urban ecological corridor (EC). To help understand the influence of land use policy on GI governance and support the optimization of spatial planning, we proposed a situation–structure–implementation–outcome (SSIO) policy cascade analysis framework. SSIO takes “place” as its starting point, then couples the local policy with the governance structure to promote the sustainability of urban commons governance. The results show that the land use type within an EC in the city is mainly cultivated land. However, between 2009 and 2019, cultivated land, construction land, and facility agricultural land all showed a decreasing trend, while forest land and garden land types underwent increasing trends. The LUC Tupu unit highlights the transition from cultivated land to forest land. Forest land has the greatest increase in area and accounts for 52.34% of the area of increasing land use. Cultivated land shows the greatest decrease in area and accounts for 70.30% of the area of decreasing trends. Based on the local policy situation of the metropolis, a land policy governance mechanism can be constructed by the establishment of a governance structure with local government as the core, using land consolidation as the platform, taking ecological spatial planning and inefficient construction land reduction as typical policy tools, and experimentally integrating the concept of Nature-based Solutions (NbS). In general, these findings may be applicable to other rapidly urbanizing cities around the world that are developing complex land use policies for ecological space governance.

## 1. Introduction

As the earth enters the Anthropocene, the earth’s ecological processes are gradually becoming dominated by human behavior, leading to catastrophic and irreversible environmental change [1,2,3]. A trend of increasing urbanization creates opportunities and challenges for human well-being and for a transition towards sustainability [4]. In order to meet these challenges and meet the United Nations Sustainable Development Goals (SDGs), government officials and researchers are looking for appropriate solutions, and Nature-based Solutions (NbS) are becoming increasingly popular [5,6,7,8,9,10,11,12,13]. NbS is an umbrella concept that includes several ecosystem-based methods such as ecosystem-based adaptation, ecosystem-based disaster risk reduction, natural climate solutions, and green infrastructure (GI) [14]. Urban green infrastructure (UGI) can be considered an NbS to urban challenges [15,16].

The spatial planning of UGI is well established in many countries [17], and UGI has developed into a crucial strategic planning tool in order to achieve sustainable urban development [18,19]. It is related to the development of urban green space, and it mainly aims at protecting biodiversity, strengthening the supply of ecosystem services, and improving the health and well-being of residents [20,21,22]. The spatial distribution and structure of UGI directly affect the function of urban green spaces [23]. However, UGI is mostly regarded as a planning and policy concept that lacks the immediacy that decision-makers pay more attention to [17]. GI and ecosystem services are closely related [24]. Some studies claim that GI should be taken into consideration when preparing spatial planning such as land use planning [25,26]. In particular, integrating GI and ecosystem services into land use planning can help enhance the health and resilience of ecosystems [27].

At present, spatial planning is facing the dual requirements of urban sustainability and flexible construction and also aims at improving urban resilience [28,29]. As currently seen in several countries, GI construction has become a key adaptation option to increase the sustainability and resilience of cities and communities. Through the implementation of multifunctional GI spatial planning, urban resilience and sustainability in post-industrial stages can be efficiently improved [26]. UGI can encompass complex and diverse urban landscape types that can include parks, public green spaces, green corridors, street trees, and roof greening [30]. 

Ecological corridors are a typical type of UGI. Ecological corridors can maintain the stability of ecosystems and can be used to limit the unplanned spread of construction land [31]. Identifying ecological corridors is of great importance for biodiversity conservation and landscape planning, and several studies have discussed relevant spatial identification and delimitation methods [32,33,34]. Identifying and delimiting the scope of an ecological corridor is only the first step in ecological corridor construction. In practice, it is more critical to carry out specific and systematic ecological space governance activities within the designated ecological corridor. The construction of ecological corridors is closely related to the policy environment of the region, especially the land governance policy. However, few studies have systematically explored land policy arrangements in the construction of ecological corridors, such as the combination of policy tools. Researchers have proposed that the observation and assessment of land use change (LUC), especially from the perspective of public policy, can be used to help regulate trends in LUC [35,36,37]. One study in south-central Chile showed that incorporating political processes into LUC and using a Dyna-CLUE modeling approach can help to understand how the policy process shapes its outcomes [38]. Spalding (2017) discussed linkages between land use management and LUC from the perspective of institutional change and explained the path to produce important local results between land management and land use [39]. Tontisirin and Anantsuksomsri (2021) analyzed the degree of LUC in the eastern region of Thailand under the influence of a Thai government project and calculated the impact of economic development policies on LUC [40]. Several studies have also examined the LUC process in a broader policy-analysis framework. For instance, Zhou et al. (2020) investigated the internal mechanism of farmland transitions using the Advocacy Coalition Framework [37]. However, existing studies have not yet systematically identified the relationship between land-governance policy and LUC related to ecological corridors. To fill this knowledge gap, this study proposes a new policy cascade analysis framework, situation–structure–implementation–outcome (SSIO), to better observe and understand the process of ecological space governance.

China’s industrialization and urbanization have accelerated rapidly since the economic reforms of 1978, and this has resulted in China currently having to face a series of related ecological problems [41]. In order to efficiently reduce or eliminate the negative impact of the deterioration of the ecological environment, the Chinese government has recently made efforts to carry out the construction of an “Ecological Civilization”. For instance, the central government in 2013 decreed that all major cities should have plans for “ecological control lines” that demarcate space between urban green space protection and urban sprawl.

As a major Chinese city, Shanghai provides a typical example of urban ecological space governance in China. Shanghai plans to construct large-scale UGIs earlier than other cities, and in recent years, Shanghai has accumulated extensive experience in UGI construction using components such as ecological corridors, country parks, and riverside greenways. Through the preparation and implementation of the Shanghai Basic Ecological Network Plan and the countryside unit plan, Shanghai specifically uses comprehensive land consolidation as a platform in order to promote the construction and governance of UGI, alongside a wealth of policy tools.

Therefore, this study uses the Shanghai Jinshan District ecological corridor as a typical example to demonstrate the situation–structure–implementation–outcome (SSIO) framework and to analyze the nexus between land use governance policy and the governance of ecological corridors. The objective of this study is to answer the following questions: (1) what are the characteristics of LUC in Shanghai’s ecological corridors under the influence of existing land use governance policies? (2) What are the mechanisms causing LUC in the ecological corridors that are the result of the land use governance policies? It is hoped that the results of the present study provide a theoretical reference for ecological space governance, for optimizing existing UGI supporting policies, and for helping to deal with urban green space development in cities around the world.

## 2. Theoretical Framework

One of the two objectives of this study is to analyze how land use governance policies cause LUC within ecological corridors. As the policy process involves multiple policy tools and governance subjects interacting at different scales, the complexity of this policy “black box” poses a challenge to researchers trying to understand the impact of the policy operation [42,43,44]. The institutional analysis and development (IAD) framework [45] and the social–ecological system (SES) framework [42] have been proposed to solve this kind of problem. However, research paradigms that explicitly guide adaptive governance practices based on a deep understanding of the process of policy operation are still limited, especially as there is a consensus among scholars that the mechanical application of existing theoretical analysis frameworks should be avoided and that local situations must not be ignored [46].

**Figure 1:**
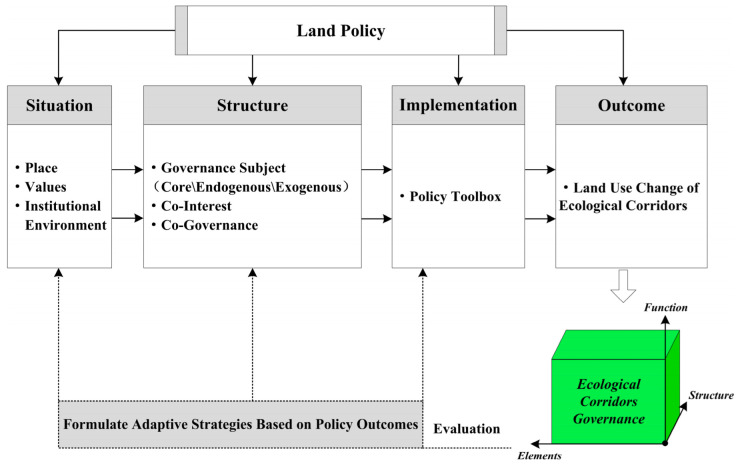
Theoretical framework: SSIO.

Therefore, in order to understand the dynamics of local situations and support the design of policies that promote adaptive governance, we propose a new policy cascade analysis framework, situation–structure–implementation–outcome (SSIO), which is characterized by taking “place” as the starting point of policy-process analysis, coupling local policy situation and governance structure, linking governance structure and policy tools, and promoting the sustainability of urban commons governance. The SSIO framework provides a comprehensive and integrated perspective that combines the policy situation, policy structure, policy implementation, and policy outcome and clarifies their complex feedback mechanisms. The SSIO framework uses the following steps to identify the relationships between land-governance policy and LUC within ecological corridors (Figure 1).

(1) Policy Situation: The operation of the policy process does not exist in a vacuum. The operation of a set of policy mechanisms is embedded in their own unique policy situation. The policy situation contains three key concepts: place, values, and institutional environment. “Place” refers to the immovable landscape of a location, including the combination of local long-term accumulated physical elements and local historical events. “Values” refers to the judgment criteria of different subjects, social phenomena, or public problems and the basic norms followed when taking specific policy actions. “Institutional environment” can be understood as a combination of existing or long-term policies in a region. Place and values interact with each other, summarized by the phrases “place shapes values” and “values influence place”. These reciprocal effects between place and values are critical to understanding the uniqueness of the institutional environment.

(2) Policy Structure: Policy structure refers to the relationship formed by the relevant policy subjects in the policy process, which reflects the power distribution pattern between the relevant policy subjects. Policy structure contains three principal concepts: governance subject, co-interest, and co-governance. The implementation of public policy depends on the interactive behavior of multiple subjects, which forms an open behavior network. Governance subjects can be divided into core governance subjects, endogenous governance subjects, and exogenous governance subjects. For example, it is most obvious in authoritarian countries such as China, where local governments are often the core governance subjects. The above three concepts are condensed into a feedback relationship, which is characterized by condensing co-interests, connecting different governance subjects, and then forming a pattern and atmosphere of co-governance.

(3) Policy Implementation: In the process of policy operation, a variety of policy tools need to be used for the implementation of policy behavior in order to promote the realization of policy objectives. Policy tools are governance techniques that help define and achieve policy objectives. Government has a “policy toolbox”, which contains different policy tools. Government chooses different policy tools according to the characteristics of the governance object so as to finally realize governance aims.

(4) Policy Outcome: Policy outcomes are often that relevant problems are solved or alleviated. For example, the policy results in this study can be expressed as the LUC of ecological corridors.

## 3. Study Area and Data

### 3.1. Study Area

The Jinshan District is one of sixteen districts in Shanghai, located in the south wing of the Yangtze River Delta and the southwest of Shanghai. According to the data of the seventh China census, the urban population of the Jinshan District in Shanghai is 506,732, accounting for 61.6% of the total Jinshan District polulation; The rural population is 316,044, accounting for 38.4%. The largest chemical industry zone in Shanghai lies in the southeast of the Jinshan District. Although the chemical industry has made Jinshan’s manufacturing industry develop rapidly, it has also produced environmental pollution. In recent years, the Jinshan District has carried out several rounds of comprehensive environmental improvement, such as vigorously implementing a reduction of inefficiently-used industrial land. At present, the Jinshan District is establishing four industrial clusters around the topics of new materials, life and health, information technology and intelligent equipment, and is striving to make these the main driving force for the economic development of the region. The Jinshan District is rich in biological resources, including more than 80 kinds of trees, nearly 10 kinds of aquatic plants, and more than 10 kinds of wild birds.

**Figure 2:**
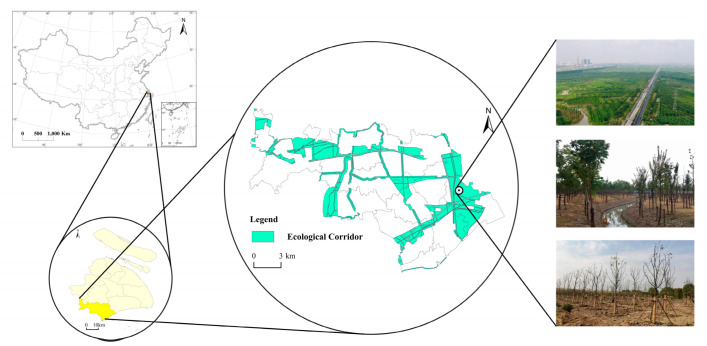
Location of the study area.

In order to protect urban green space, the Shanghai Municipal Government formulated the Shanghai Basic Ecological Network Plan in 2010, which is the main component of Shanghai’s ecological policy and lays the foundation for the delimitation of ecological corridors. According to the Shanghai Land Consolidation Plan (2016–2020) and the Shanghai Ecological Corridors System Plan (2017–2035), the total area of the Shanghai ecological corridors is 2476.46 km^2^. The ecological corridor in the Jinshan District is a typical example of Shanghai ecological corridors. The total area of ecological corridors in the Jinshan District is 160.30 km^2^, which accounts for 27.35% of the total land area in the Jinshan District (Figure 2).

### 3.2. Data Source and Processing

**Figure 3:**
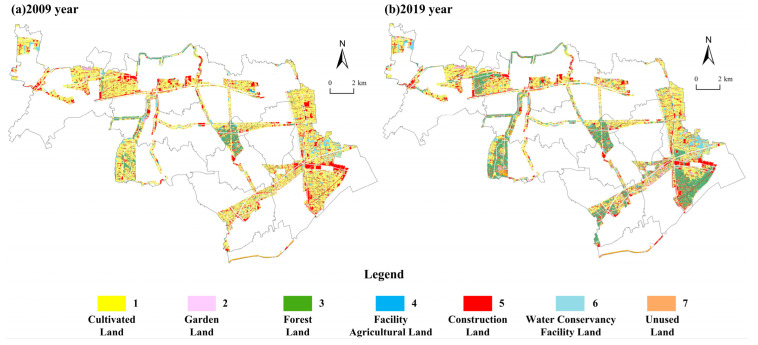
Land use status in 2009 and 2019. (Note: Nos. 1–7 represent cultivated land, garden land, forest land, facility agricultural land, construction land, water conservancy facility land, and unused land.)

The land use data used in this study was vector data of land use status in the Shanghai Jinshan District for the years 2009 and 2019, provided by the Shanghai Municipal Bureau of Planning and Natural Resources. Data processing consisted of the following steps: (1) Using ArcGIS 10.3 software, the raw land use data were mask-segmented in order to obtain a land use spatial database for the study years. (2) Using the classification standard of land use status (GB/T21010-2017), all land use types were divided into seven standardized types, i.e., cultivated land, garden land, forest land, facility agricultural land, construction land, water conservancy facility land, and unused land. Among these, garden land refered to the land where fruit trees, tea trees, medicinal materials, and other perennial crops are planted. For these land use types, land use coded values were set to 1, 2, 3, 4, 5, 6, and 7, respectively (Figure 3). (3) With the help of conversion tools in the ArcToolbox module of ArcGIS 10.3, the vector data of land use status code values in the ecological corridors of Jinshan District for 2009 and 2019 were converted to 10 m × 10 m gridded data. (4) Geo-informatic Tupu analysis, land use dynamic degree analysis, and fluctuation potential atlas analysis were conducted to analyze the characteristics of LUC in the ecological corridors of the Jinshan District.

## 4. Methods

### 4.1. Geo-Informatic Tupu Analysis

The geo-informatic Tupu method is a type of geographical spatial-temporal analysis method that is commonly used to reveal the internal structure and spatial differentiation of geographical elements [47]. Additionally, the geo-informatic Tupu analysis method can reveal the spatio-temporal change in the LUC using the Tupu unit. The Tupu unit is the fundamentally functional unit in integrated spatial-temporal analysis of land use [47]. As with the basic geographical unit, the division of the relatively homogeneous Tupu unit is also very important for such research [48]. The Tupu units of LUC in this study were summarized into one type, that is, land use type changes from 2009 to 2019. For example, Code 15 is a Tupu unit, which represents cultivated land in 2009 changed to construction land in 2019, and other codes follow the same rules.

To build the Tupu process for the LUC, in this study, the algebraic superposition of the Tupu unit for LUC was conducted within the ArcGIS 10.3 software package, which was used to integrate the spatial information of the Tupu-coded LUC values [49]. The codes of adjacent cells in the gridded data were selected for the algebraic operation to obtain the LUC Tupu value. The equation is as follows:

C=10×A+B

where *C* represents the Tupu code of the LUC during the study period, *A* represents the land use unit code value at the beginning of the study period, and *B* represents the land use unit code value at the end of the period. Using these steps, the LUC Tupu values for the ecological corridors of the Jinshan District in Shanghai from 2009 to 2019 were obtained.

### 4.2. Fluctuation Potential Tupu Analysis

LUC describes the process of changes in land use from one stable form to another, driven by factors such as economic and social development over a specific period. The process of LUC includes transfer-in and transfer-out levels. Transfer-in refers to the transfer of other land use types to the target land use type, which results in an increase in the area of the target land use type and hence results in an increasing areal trend. Transfer-out refers to the conversion of the target land use type to other land use types, which results in the shrinkage of the area of the target land use type and hence a decreasing areal trend. Based on superposition analysis and the generation of Tupu land use data for 2009 and 2019, using ArcGIS 10.3, the increase and decrease in the areas of certain land use types were obtained, and maps of increasing (land use rising trend maps) and decreasing (land use falling trend maps) areal trends were constructed.

## 5. Results

### 5.1. Characteristics of LUC from 2009 to 2019

#### 5.1.1. Analysis of Land Use Structure Change

**Figure 4:**
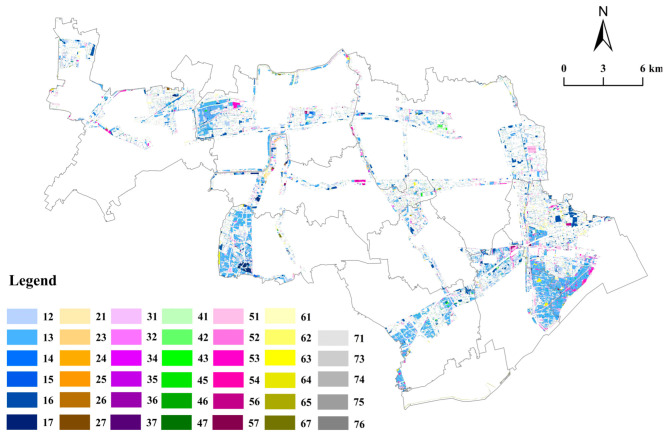
LUC of ecological corridors in the Jinshan District. (Note: Nos. 1–7 represent cultivated land, garden land, forest land, facility agricultural land, construction land, water conservancy facility land, and unused land, respectively. Code 15 represents cultivated land converted to construction land, and other codes follow the same rule.)

It can be seen from Figure 4 and Table 1 that the land use structure change in the ecological corridors of the Jinshan District presents five different characteristics: ① The main land type of the ecological corridors of the Jinshan District was that of cultivated land. Cultivated land area exceeded 40% for both 2009 and 2019. However, with the high speed of industrialization and urbanization, the pressure on cultivated land remained high, and the area of this land type showed a decreasing trend. The proportion of cultivated land in the total area of the study area decreased from 62.46% in 2009 to 42.87% in 2019, with an average annual change rate of −3.14%. ② Benefiting from the development of modern agriculture, as well as the continuous expansion of orchard area used for both tourism and fruit production, the area of the garden land type in the ecological corridors of the Jinshan District increased over the 10-year study period, from 119.41 hm^2^ in 2009 to 469.11 hm^2^ in 2019, with an average annual change rate of 29.29%. ③ Compared with the other land types, the growth in the forest land area was the most obvious. Its proportion in the total area of the region significantly increased, with an average annual change rate of 36.37%. This indicates the efficiency of the policy of returning farmland to forest, reducing inefficient construction land, and the success of the guidance provided by the ecological corridors of the Jinshan District construction plan. ④ With the rapid growth in forest land area and the continuous reduction in cultivated land area, the growth space for facility agricultural land was suppressed to a certain extent, and its area decreased from 321.19 hm^2^ in 2009 to 71.63 hm^2^ in 2019, with an average annual rate of change of −7.77%. ⑤ The total amount of construction land shrank over the study period. It decreased from 3196.38 hm^2^ in 2009 to 2900.69 hm^2^ in 2019, with an average annual change rate of −0.93%. This indicates that the policy of reducing inefficient construction land outside urban centralized construction areas achieved remarkable results in curbing the inefficient growth of construction.

#### 5.1.2. Tupu Analysis of LUC from 2009 to 2019

From 2009 to 2019, the LUC Tupu analysis generated 48 types of Tupu units, of which 41 types changed the land use types (Figure 4). The Tupu units are ranked according to their area (Table 2) and their calculated change rates are also presented. It can be seen that the cumulative decadal change rate of 18 kinds of Tupu units reaches 97.03%, revealing several different spatial distributions. ① The transition of cultivated land use occupied a dominant position, undergoing areal contraction and function optimization to a certain extent. The Tupu unit identifying “cultivated land → forest land” (Code 13) is the most common. From 2009 to 2019, the LUC Tupu analysis mainly reveals the conversion of cultivated land to forest land, with a rate of change of 44.83%, the conversion of cultivated land to water conservancy facility land with a rate of change of 9.63%, and the mutual conversion of cultivated land with construction land (i.e., the rate of change of construction land to cultivated land was 8.34%, while that of cultivated land to construction land was 7.06%). Cultivated land was mainly converted to forest land, water conservancy facility land, construction land, and garden land. The relevant transfer-out areas were 2530.45 hm^2^, 543.56 hm^2^, 398.34 hm^2^, and 375.10 hm^2^, respectively, with a cumulative transfer-out ratio of 68.17%. The coupling effect of ecological conversion of farmland, water conservancy construction, construction occupation, and tourist park construction were the main reasons for the shrinkage of the cultivated land area during the study period. ② Driven by the policy of reducing inefficient construction land, the structural and functional transition of construction land has been preliminarily achieved. Because of the reduction in inefficient industrial land and the renovation of rural residential areas in the ecological corridors of the Jinshan District, the area of construction land significantly shrank. Inefficient construction land was mainly converted and reclaimed into cultivated land and forest land, resulting in an increase in the cultivated land area and the forest land area.

#### 5.1.3. Analysis of Land Use Fluctuation Potential Map

##### Land Use Rising Trend Map

**Figure 5:**
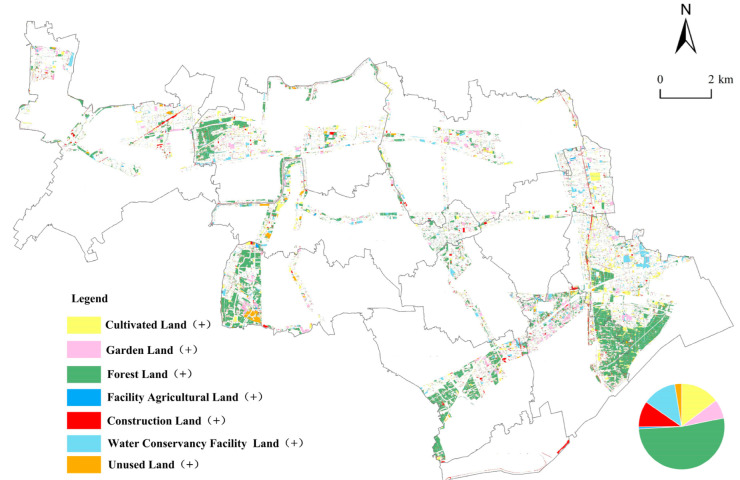
Rising graphics of land use in the ecological corridors of Jinshan District. (Note: the legend “cultivated land (+)” represents the spatial distribution of other land types transformed into cultivated land, and other legends follow the same rules.)

Using the rising trend map analysis method, the rising trend map of the time series units of ecological corridors from 2009 to 2019 was generated (Figure 5, Table 3). ① From 2009 to 2019, the newly added forest land area was the largest area showing a rising trend. It accounted for 52% of the areas with a rising land use trend, which is consistent with the policy goal and governance direction in the ecological corridors of the Jinshan District. ② Newly added forest land was followed by new cultivated land, new water conservancy facility land, new construction land, and new garden land, having areas of 826.81 hm^2^, 720.4 hm^2^, 552 hm^2^ and 403 hm^2^, respectively, which accounted for 14.65%, 12.76%, 9.78%, and 7.15% of the area change. ③ In terms of sources of land use types increasing in the area, the sources of new cultivated land were mainly construction land and water conservancy facility land, accounting for 56.94% and 25.64% of the total area of the new cultivated land sources, respectively. The main source of areas of new garden land was cultivated land, which accounted for 92.97% of the total area of new gardens. The main sources of new forest land were cultivated land and construction land, accounting for 85.67% and 8.81% of the total rising area of forest land, respectively. Sources of new facility agricultural land were mainly cultivated land and construction land, accounting for 86.76% and 8.38% of the total area of the facility agricultural land, respectively. The sources of new construction land were mainly cultivated land, water conservancy facility land, and facility agricultural land, accounting for 72.20%, 12.45%, and 8.83% of the total rising area of the construction land, respectively. Sources of new water conservancy facility land were mainly cultivated land, facility agricultural land, and construction land, accounting for 75.49%, 13.05%, and 9.36% of the total area of the water conservancy facility land, respectively. Sources of new unused land were mainly cultivated land, construction land, and water conservancy facility land, accounting for 57.47%, 21.62%, and 13.09% of the total area of unused land, respectively.

##### Land Use Falling Trend Map

**Figure 6:**
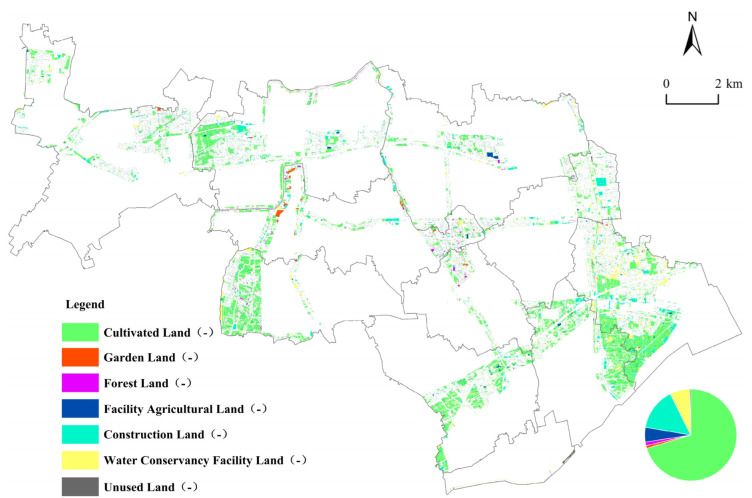
Falling graphics of land use in the ecological corridors of Jinshan District. (Note: the legend “cultivated land (-)” represents the spatial distribution of cultivated land transformed into other land types, and other legends follow the same rules.)

Using the falling trend map analysis method, the falling trend map of time series units of ecological corridors from 2009 to 2019 was generated (Figure 6). From 2009 to 2019, the area of cultivated land showing a falling trend in the study area was the highest (3967.66 hm^2^), accounting for 70.30% of the falling trend of the changed area. The Law of the People’s Republic of China on Land Administration stipulates that China implements a cultivated land compensation system. This means that a corresponding amount of cultivated land needs to be increased in the external area of the ecological corridor when the cultivated land area inside the ecological corridor decreases. Therefore, the pressure of cultivated land protection will be transmitted from the inside of the ecological corridor to the outside of the ecological corridor. The area of reduced construction land was 847.38 hm^2^, accounting for 15.01% of the falling trend change area. The area of reducing water conservancy facility land was 389.20 hm^2^, accounting for 6.90% of the changed area. The area with reducing facility of agricultural land was 290.95 hm^2^, accounting for 5.16% of the changed area. The area with reduced forest land was 71.88 hm^2^, accounting for 1.27% of the changed area. The source of the falling trend of cultivated land was mainly forest land, which accounts for 63.8% of the total area of cultivated land with a falling trend. The falling trend of construction land reveals that construction land was mainly transformed into cultivated land and forest land, which account for 55.55% and 30.72% of the total area of the construction land, respectively. The falling flow direction of construction land once again reveals an implementation effect of the reduction in inefficient construction land in this region.

### 5.2. Land Policy Governance Mechanism of Ecological Corridors

#### 5.2.1. Policy Situation

The role of values, norms, and principles in governance practice is usually vague. However, it constitutes the basis of all practical policy-based decisions and inspires managers to think, judge, and take actions. For example, SDGs aim at solving the conflict between the two values of “development” and “sustainability” by trying to make the two values compatible with each other. Values and the institutional environment will also be affected by the local characteristics of a region.

According to the overall urban plan for Shanghai (2017–2035), Shanghai aims to build a more sustainable and resilient ecological city. It will specifically build a multi-level, networked, and functional urban ecological space system composed of “double rings, nine corridors and ten districts”. The construction of ecological corridors is an important measure for Shanghai to realize urban sustainable development, improve the resilience of the urban spatial structure, and promote investment and construction under UGI. This policy action reflects the value cognition of Shanghai urban managers.

A close match exists between the goal and vision of the construction of ecological corridors and comprehensive land consolidation. As an efficient measure for adjusting and optimizing the economic and social development of a metropolis, comprehensive land consolidation plays a crucial role in shaping high-quality land space and coordinating the relationship between development and protection. In order to stimulate and maintain the instrumental role of comprehensive land consolidation in the allocation of resources, Shanghai has explored the “land consolidation +” model. “Land consolidation +” is a high-level form of land consolidation, which shows that it pays more attention than other forms to the driving role of land consolidation in attracting industries, integrating funds, and promoting ecological environment restoration [50]. In practice, models such as “land consolidation + field art”, “land consolidation + multifunctional agriculture”, and “land consolidation + country park” are emerging.

#### 5.2.2. Policy Structure

Policy structure mainly involves multiple policy subjects and their strategic interaction. In Shanghai, these are represented as follows.

**Figure 7:**
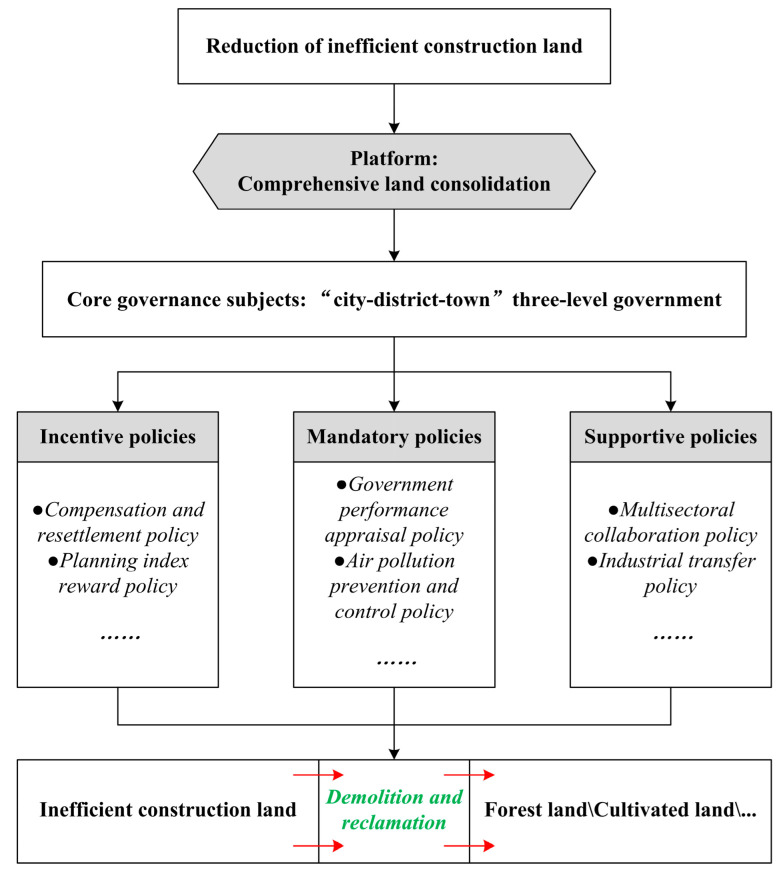
Implementation mechanism of inefficient construction land reduction.

(1) Multiple policy subjects: The LUC of ecological corridors is a typical geographical change process in a regional system. The key policy subjects involved in this process include the three-level city–district–town local government, enterprises, farmers’ collectives, experts, researchers, and social media. The three-level city–district–town government is the core governance subject. By establishing a multi-level policy linkage promotion mechanism, local governments in the Shanghai region have built an institutional system for comprehensive land consolidation, forming a policy guarantee system to promote the protection and restoration of ecological corridors. Farmers and village committees are endogenous governance subjects, which are the basic forces involved in promoting comprehensive land consolidation within Shanghai. Enterprises are typical subjects of exogenous governance. In addition, other exogenous governance subjects, such as experts, researchers, and social media, have also participated in the process of comprehensive land consolidation in the whole Shanghai region.

(2) Strategic interaction of policy subjects: The motive force of the strategic interaction of different policy subjects mainly comes from competition and games of interest. Change and stability of the benefit distribution pattern directly affect the direction and rate of LUC. For example, Shanghai has established a policy support and financial subsidy system to reduce inefficient construction land (Figure 7). Shanghai Municipal Finance will subsidize the specific plots of inefficient construction land reduction according to the corresponding standards. Local governments are building a fair benefit-sharing mechanism, developing ordered rules of green space governance, maintaining a benign interaction with the policy structure, and ensuring that the LUC of ecological corridors in Shanghai is coherent with regional planning visions and social objectives.

#### 5.2.3. Policy Implementation

Policy implementation requires the help of multiple policy tools to transform policy objectives into policy outcomes. The completeness of policy tools determines the combination mode and implementation effect of policy behavior. It also determines the efficiency and order of different subjects participating in co-production and co-creation. The premise of the choice and application of government tools consists in identifying and classifying policy tools. The classification of policy tools mainly focuses on two categories: “mandatory” and “non-mandatory” tools. Based on existing studies [51,52], this study classifies the policy tools used in the Shanghai region into four levels: regulatory, economic, and organizational policy tools.

(1) Organizational policy tools: These mainly include general consolidation and reclamation, municipal land consolidation projects, country park construction, and the reduction in inefficient industrial land directly promoted by government agencies. The reduction in inefficient construction land refers to the reduction in and reclamation of inefficient construction land in rural areas into cultivated land or ecological land by using policy measures and engineering technology. The policy tools used in the reduction in inefficient construction land can be divided into incentive policies, mandatory policies, and supportive policies (Figure 7). In addition, comprehensive land consolidation activities are widely carried out in the ecological corridors of the Jinshan District. With the help of comprehensive land consolidation, country parks in the ecological corridors of the Jinshan District realize the reduction and reclamation of rural inefficient industrial and mining land, homestead, and other construction land and focus on the management of fields, water, roads, and forests. The Basic Ecological Network Planning of Shanghai stipulates that large forests can be built within ecological corridors, in combination with the protection of basic farmland, and country parks can be arranged at important nodes. This provides a planning basis for the layout of country parks at appropriate nodes within ecological corridors, which will help promote UGI investment.

(2) Economic policy tools: These mainly include taxation, determination and transaction of property rights, appropriation, and the conclusion of contracts and subsidies. In order to accelerate the orderly withdrawal of inefficient construction land in the ecological corridors of the Jinshan District and improve the transformation rate of inefficient construction land, Shanghai has developed and implemented a reduction in inefficient construction land outside the centralized construction area since 2014. However, the capital demand for inefficient construction land reduction is huge, the cost of reclamation is very high, and the process is complex. Therefore, Shanghai has developed a special fund to support the reduction in inefficient industrial land.

(3) Regulatory policy tools: These mainly include management regulations, organizational leadership, and system planning. For instance, Shanghai has gradually developed a strict land-use and space-use control system by formulating land consolidation planning, ecological-network planning, and rural-unit planning. These form a whole process control order in the ecological corridors of the Jinshan District.

#### 5.2.4. Policy Outcome

Land use within the ecological corridors has changed from “mixed” to “ecological”. This process has mainly been achieved by integrating the objectives and vision of urban sustainable and resilient development into the whole process of policy formulation and implementation while optimizing the interactive structure of policy subjects using combined policy tools. The main results are summarized as follows. 

With the help of regulatory policy tools, local governments have developed institutional arrangements of ecological corridors construction and governance so as to lay the foundation for the transformation of land use within ecological corridors.

The basic governance logic has been to integrate a variety of policy tools, manage ecosystem functions, mitigate ecosystem risks, and then implement ecological space adaptive governance. To effectively supplement the area of cultivated land and forest land within the ecological corridors, Shanghai has built a set of land governance policies. By implementing the reduction in inefficient construction land, “zombie” enterprises located in the planning scope of ecological corridors were intensively eliminated. Industries with a scattered spatial distribution and efficiency characteristics termed “three high and one low” (i.e., high pollution, high energy consumption, high investment, and low efficiency) were also eliminated (Figure 7). In addition, this was achieved by taking comprehensive land consolidation as the platform, linking inefficient construction land reduction and country park construction, improving the orderly exit mechanism of inefficient construction land, and implementing ecosystem protection and restoration.

## 6. Discussion

### 6.1. Interpretation of Land Use Change Characteristics

Figure 4, Figure 5 and Figure 6 show the LUC within the ecological corridors over the past 10 years (i.e., from 2009 to 2019), of which the most obvious and drastic changes are to cultivated land, forest land, and construction land. The relationship between the conversion of cultivated land and construction land has been studied extensively [53,54]. In general, the strong demand for construction land can reduce cultivated land area, which makes the areas of construction land and cultivated land show opposite trends. For instance, Yin et al. (2020) documented an apparent decrease in cultivated land, with an associated increase in construction land in the Yellow River Basin from 1990 to 2018 [55]. Chen et al. (2020) found that the Yangze River Economic Belt in China of construction land continued to markedly increase, while the area of cultivated land continuously decreased from 1995 to 2015 [56]. In this study, cultivated land area in the ecological corridors decreased from 10,012 hm^2^ in 2009 to 6871 hm^2^ in 2019. Construction land area also decreased from 3196 hm^2^ in 2009 to 1836 hm^2^ in 2019. The area of the cultivated land and construction land both showed shrinking trends, which is different from the results obtained by other studies. A possible reason for the shrinkage in construction land is the policy of the reduction in industrial land beyond the Urban Development Boundary applied in Shanghai since 2014 [57,58]. During the implementation of Shanghai’s industrial land-reduction policy, priority was given to reducing industrial and enterprise-related land with high investment, high energy consumption, and high pollution but low efficiency, as well as low-efficiency industrial land in areas such as farmland protection areas, water source protection areas, ecological network spaces, and planned country parks [57]. Under the influence of this policy, inefficient construction land was reclaimed and converted into cultivated land and forest land [59]. The results of this study verify that the main source of the rise in cultivated land came from construction land, which accounts for 56.9% of the total area of the rise in cultivated land, as shown in Table 3.

Compared with the reduction in cultivated land and construction land, the growth of the forest land area is obvious. The proportion of forested area to the total area of the ecological corridor increased from 4.9% in 2009 to 22.9% in 2019. The Jinshan District has always contained an important petrochemical industry cluster in Shanghai. In recent years, the region has carried out a large-scale action of returning farmland to forest in order to reduce the air pollution and soil pollution caused by chemical enterprises. In addition, with the increasing demand of urban residents for green ecological space, country parks have gradually become a new planning choice in order to meet the needs of residents for ecological services and recreational facilities [41]. Several studies have shown that the natural landscape of country parks of a large size is more attractive than semi-natural landscapes, such as farmland, for example [41]. Countryside forest is a crucial resource to meet the growing outdoor recreational needs of urban residents [60,61]. According to basic ecological network planning within Shanghai, country parks are planned and built inside ecological corridors. Therefore, the ecological and economic benefits of returning farmland to forest are emerging, accompanied by the construction and operation of country parks.

### 6.2. Policy Supply and Ecological Space Governance

With the increasing demand for ecological space, ecological space governance has become a crucial topic [22]. Several studies emphasize the importance of ecological space governance [8,10,62,63]. For instance, Sterner et al. (2019) believe that the design of policy and of the related governance structure should be seriously considered to deal with the risks of overstepping “planetary boundaries” [64]. Arjen et al. (2018) believe that the role of discourse, resource, actors, and rules of the game are crucial in the upscaling of active citizenship [65]. Møller et al. (2019) studied how e-tools can support UGI governance and perceived related barriers. They also demonstrated that place-based e-tools have potential for UGI governance [63].

This paper agrees that enriching the governance policy toolbox will enhance the comprehensive benefits and value of green infrastructure such as ecological corridors, for example. In addition, in terms of the choice of valuable policy tools in Shanghai, NbS has developed into an important set of methods to deal with urban challenges [12,66]. NbS particularly emphasizes the value of nature in addressing urban challenges [67], as well as the common interests of society, ecology, economy, and behavior [16]. However, NbS still has difficulties in mainstreaming urban land governance policies [68], although they have been valued in some European cities, such as London [69]. According to the investigation presented in this study, it can be demonstrated that, although the term “NbS” has not been mainstreamed in Shanghai’s ecological space governance policies, some policies that have been issued have shown the use and attention to nature. The policy of reducing inefficient industrial land in Shanghai requires that inefficient industrial sites outside the urban development boundary should withdraw in an orderly manner and ecological reclamation should be performed. In addition, it was necessary to perform soil-quality evaluation on reclaimed cultivated land that has met farming requirements.

Rigid land use management and flexibility are integrated into whole-life-cycle management [70]. Through comprehensive land consolidation and ecological restoration, the spatial layout of land elements can be optimized and the supply and demand flow of ecosystem services can be regulated. By promoting the construction of farmland-forest networks, the regulatory role of forest land on farmland and water system ecosystems is achieved. Some studies have revealed that the implementation of comprehensive land consolidation policies is helpful for value visualization and dynamic allocation of multi-function cultivated land [71]. The planning of Shanghai’s ecological space (2021–2035) demonstrates that priority should be given to the layout of country parks in ecological corridors. Although both country parks and urban parks are designed to meet the ecological and recreational needs of residents, they show clear differences in planning, construction, and operation [41]. At present, Shanghai is in the post-industrialization stage, and traditional urban parks can no longer meet the needs of citizens for open green space. Shanghai’s country parks are mainly based on existing large areas of farmland, villages, and forests, emphasizing the multi-functional utilization of the natural landscape. However, as the recreational attraction of metropolitan suburban ecological space to urban residents is increasing, it is also necessary to prevent the migration of pollutants, such as vehicle exhaust. Therefore, in order to better balance the contradiction between residents’ leisure needs and traffic pollution, it is also necessary to develop public transport networks, including establishing public transportation sites near country parks [41].

### 6.3. Developing a New Policy Cascade Analysis Framework

**Figure 8:**
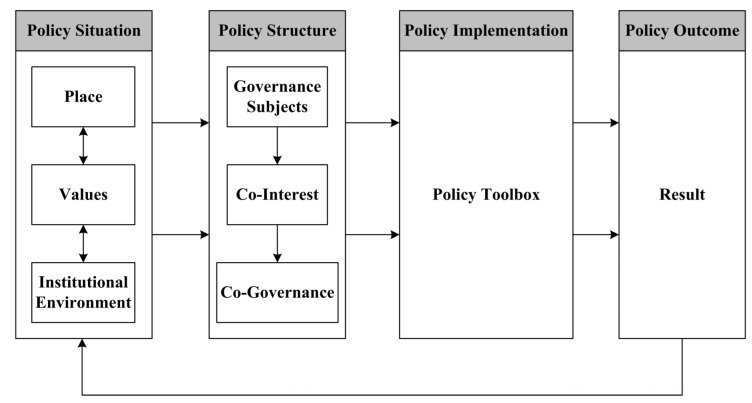
SSIO: a policy process analysis framework.

The SSIO policy analysis framework developed in this study is mainly applicable to observing the implementation process of a specific policy. In order to increase the applicability of the analysis framework, its relationship, based on Figure 1, is further described (Figure 8). If other researchers want to use this analytical framework, they need to pay attention to the following aspects.

(1) Understanding the internal relationship between locality and historicity. This study believes that “place” should be the beginning of understanding a policy process. This is due to the fact that the one-size-fits-all regional policy models may not be successful and should be replaced by place-specific and knowledge-based policies [46]. The identification of challenges and their potential solutions requires the use of knowledge of regions and local communities. Place refers to the characteristics of a place that are different from other places. Traits cannot be completely replicated elsewhere. For instance, urban areas are hybrid systems where natural and artificial components, as well as social and ecological processes, are blurred and interconnected [72]. Framing such strategies and interventions with specific solutions requires a good understanding of the local environmental, social, and economic conditions in the urban context [66]. Place is mainly composed of three aspects: local immovable landscape elements, the combination of physical elements locally accumulated for a long time, and local historical events.

(2) Understanding the interaction between place and values. Values are the basis for individuals to understand things, distinguish right from wrong, and make decisions. They are relatively stable, persistent, and subjective. The formation of local values can be reflected in the vision and objectives of urban development, as well as the choice of urban governance tools.

(3) The universality of structure. Many disciplines pay attention to structure. For instance, sociology is good at observing the structural characteristics of society and attributing the problems and difficulties a country is experiencing to the social structure. In addition, geography is good at observing the human–Earth areal system from the perspective of element–structure–function. In the field of public policy, the understanding of the policy structure should be based on the interactive relationship of governance subjects. Moreover, the choice of specific policy tools should also be based on the cognition of the policy situation and the policy structure, which will determine the implementation effect of policy tools.

## 7. Conclusions

Based on multi-source data, with the help of the geo-informatic Tupu analysis, fluctuation potential atlas analysis, and the construction of a new SSIO policy process analysis framework, this paper analyzes the LUC’s characteristics and policy operation mechanism of the ecological corridors in the Jinshan District of Shanghai. The main conclusions are summarized as follows.

The land use structure within the ecological corridors is still dominated by cultivated land. From 2009 to 2019, although cultivated land area maintained a high proportion, it was affected by economic development, forest construction, and other factors and hence showed a shrinking trend, and pressure on cultivated land protection remains high. The proportion of construction land shrank, while that of forest land increased, which is consistent with the basic policy direction of the ecological corridors.

From 2009 to 2019, the LUC Tupu of the ecological corridors mainly highlighted the conversion of cultivated land to forest land, water conservancy facility land, construction land, and garden land. Since the beginning of the 21st century, China has implemented the policy of returning farmland to forests, which is mainly to restore the cultivated land with a high slope that easily causes soil erosion to forest vegetation. The policy of returning farmland to forests supports the conversion of cultivated land to forest land in the ecological corridor. High-yield farmland needs the support of perfect water conservancy facilities. Driven by the policy of high-standard farmland, a certain amount of cultivated land has been converted into land for water conservancy facilities. During this period, a certain amount of cultivated land was still converted to construction land, which shows that the contradiction between cultivated land protection and economic construction is still prominent. In recent years, China has strictly controlled the conversion of cultivated land use, especially from cultivated land to garden land and other land types. The newly revised land administration law of the people’s Republic of China has also made strict provisions for this. However, there is still a certain amount of cultivated land converted into garden land, which indicates that the phenomenon of non-grain conversion of cultivated land is relatively serious. During the study period, construction land was mainly transformed into cultivated land and forest land, which was due to the reduction in inefficient construction land. From 2009 to 2019, in the rising trend map of land use within the Jinshan ecological corridor in Shanghai, the area of newly added forest land was the largest, followed by that of newly added cultivated land, newly added water conservancy facility land, newly added construction land, and newly added garden land. In the falling trend map, the area of cultivated land in the study area decreased the most, followed by the area of construction land, and then by the area of water conservancy facility land. The area of unused land decreased the least.

With the help of the SSIO policy process analysis framework, this study analyzes the driving mechanism of land governance policy on the LUC within ecological corridors. This method can demonstrate the process of occurrence and organizational relationship of LUC in a panoramic way. Defining a local context, developing an organizational structure, selecting policy tools, and realizing adaptive governance are the four stages of systematically understanding the policy driving mechanism of LUC within ecological corridors. Finally, the proposed framework can be used to help to deconstruct the policy black box of ecological space governance and to better understand the process of ecological space governance.

## Figures and Tables

**Table 1:** LUC within the ecological corridors of the Shanghai Jinshan District from 2009 to 2019.

Land Use Type	2009		2019		Change Percentage (%)
	Area (hm^2^)	Proportion (%)	Area (hm^2^)	Proportion (%)	2009–2019
Cultivated Land	10,012.10	62.46%	6871.20	42.87%	−3.14
Garden Land	119.41	0.74%	469.11	2.93%	29.29
Forest Land	792.36	4.94%	3674.36	22.92%	36.37
Facility Agricultural Land	321.19	2.00%	71.63	0.45%	−7.77
Construction Land	3196.38	19.94%	2900.69	18.10%	−0.93
Water Conservancy Facility Land	1505.15	9.39%	1835.82	11.45%	2.20
Unused Land	83.17	0.52%	206.71	1.29%	14.85
Total	16,029.76	100%	16,029.52	100.00%	——

**Table 2:** The order of the main geographic unit of LUC in the study area.

Sequence	Type	Area (hm^2^)	Change Ratio (%)	Cumulative Change Ratio (%)
1	13	2530.45	44.83%	44.83%
2	16	543.56	9.63%	54.47%
3	51	470.76	8.34%	62.81%
4	15	398.34	7.06%	69.86%
5	12	375.10	6.65%	76.51%
6	53	260.31	4.61%	81.12%
7	61	211.98	3.76%	84.88%
8	46	93.95	1.66%	86.54%
9	63	84.82	1.50%	88.05%
10	17	84.30	1.49%	89.54%
11	41	78.40	1.39%	90.93%
12	65	68.71	1.22%	92.15%
13	56	67.37	1.19%	93.34%
14	43	59.39	1.05%	94.39%
15	45	48.69	0.86%	95.25%
16	14	35.91	0.64%	95.89%
17	31	32.86	0.58%	96.47%
18	57	31.72	0.56%	97.03%

Note: Nos. 1–7 represent cultivated land, garden land, forest land, facility agricultural land, construction land, water conservancy facility land, and unused land, respectively. Code 15 represents cultivated land converted to construction land, and other codes follow the same rule.

**Table 3:** The structure of fluctuation graphics of land use in the study area (hm^2^).

Land Use Types	Rising Area	Rise Source	Falling Area	Fall Source
Cultivated land	826.81	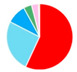	3967.66	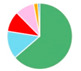
Garden land	403.46	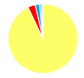	53.75	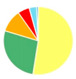
Forest land	2953.88	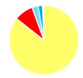	71.88	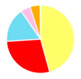
Facility agricultural land	41.39	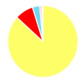	290.95	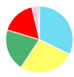
Construction land	551.70	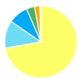	847.38	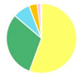
Water conservancy facility land	720.04	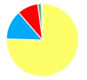	389.20	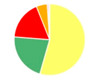
Unused land	146.69	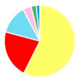	23.15	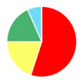

Notes: 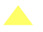 Cultivated land; 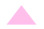 Garden land; 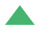 Forest land; 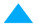 Facility agricultural land; 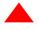 Construction land; 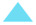 Water conservancy facility land; 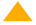 Unused land.

## Data Availability

Not applicable.
